# Extracorporeal membrane oxygenation in *Pneumocystis jirovecii* pneumonia: outcome in HIV and non-HIV patients

**DOI:** 10.1186/s13054-019-2661-9

**Published:** 2019-11-14

**Authors:** Jonathan Rilinger, Dawid L. Staudacher, Siegbert Rieg, Daniel Duerschmied, Christoph Bode, Tobias Wengenmayer

**Affiliations:** 1 Department of Medicine III (Interdisciplinary Medical Intensive Care), Medical Center, Faculty of Medicine, University of Freiburg, Freiburg, Germany; 2 Department of Cardiology and Angiology I, Heart Center Freiburg University, Faculty of Medicine, University of Freiburg, Hugstetterstr 55, 79106 Freiburg, Germany; 3 Division of Infectious Diseases, Department of Medicine II, Medical Center – University of Freiburg, Faculty of Medicine, University of Freiburg, Freiburg, Germany

**Keywords:** Extracorporeal membrane oxygenation, *Pneumocystis jirovecii*, Acute respiratory distress syndrome, Outcome

## Main text

*Pneumocystis jirovecii* pneumonia (PJP) is a severe complication of immunosuppression that is associated with high mortality, depending on the underlying type of immunosuppression [1]. Consequently, the incidence of PJP is higher in non-HIV patients than in HIV patients, because of the increased use of immunosuppressive therapies for widespread indications [2]. So far, there is little evidence for veno-venous extracorporeal membrane oxygenation (ECMO) treatment in cases of PJP-induced severe acute respiratory distress syndrome (ARDS). Particularly, there is no study reporting and comparing the outcome of PJP requiring ECMO therapy in HIV and non-HIV patients.

Therefore, we report retrospective data of a single-centre registry of patients with severe respiratory failure, requiring ECMO support at our centre between January 2009 and April 2019. ECMO support was initiated when lung-protective mechanical ventilation was not able to prevent hypoxemia or hypercapnia, based on the treating medical team’s judgement.

A total of 337 ECMO patients were screened, and 18 patients with PJP were identified (Table 1). Diagnosis of PJP was verified via positive immunofluorescence microscopy in 13 patients (72%). Five patients (28%) displayed high PCR levels (median 67.000 [5.200–250.000] copies/ml) with conclusive symptoms and radiological findings but negative immunofluorescence microscopy. Microbiological testing was performed in bronchoalveolar lavage. In 14 patients (78%), PJP was diagnosed before the initiation of ECMO therapy.

**Table 1:** Baseline characteristics and outcome

	All (*n* = 18)	HIV (*n* = 6)	non-HIV (*n* = 12)	*p* value
Age (years)	49.7 ± 18.4	36.8 ± 9.7	56.2 ± 18.6	0.032
Sex (male)	11 (61.1%)	4 (66.7%)	7 (58.3%)	1.0
BMI (kg/m^2^)	24.6 ± 3.4	23.0 ± 4.2	25.5 0 ± 2.6	0.149
Underlying pulmonary disease*	2 (11.1%)	0 (0%)	2 (16.7%)	0.407
Comorbidities				
Hypertension	5 (27.8%)	0 (0%)	5 (41.27%)	0.114
Renal insufficiency	2 (11.1%)	0 (0%)	2 (16.7%)	0.529
Chronic haemodialysis	1 (5.6%)	0 (0%)	1 (8.3%)	1.0
MV pre-ECMO				
PEEP (mbar)	14.9 ± 3.1	13.8 ± 2.9	15.3 ± 3.2	0.489
Plateau pressure (mbar)	28.5 ± 4.6	29.3 ± 4.0	28.2 ± 4.9	0.571
Driving pressure (mbar)	13.6 ± 4.2	15.5 ± 4.5	12.9 ± 4.1	0.412
Tidal volume (ml)	390.7 ± 107.9	362.5 ± 104.4	400.9 ± 112.3	0.571
Minute volume (l/min)	9.9 ± 3.6	10.6 ± 4.3	9.6 ± 3.5	0.571
Compliance (ml/mbar)	32.7 ± 15.8	23.3 ± 10.4	35.5 ± 17.0	0.226
FiO_2_ (%)	83.8 ± 19.4	87.5 ± 19.4	81.8 ± 19.4	0.660
Horowitz index (mmHg)	87.6 ± 37.6	90.8 ± 40.8	85.8 ± 37.6	1.0
D(A-a)O_2_ (mmHg)	466.4 ± 133.4	481.7 ± 132.9	458.1 ± 139.4	0.884
MV duration before ECMO (days)	5.4 ± 5.4	9.3 ± 6.5	3.3 ± 3.3	0.048
Acute renal failure	3 (16.7%)	0 (0%)	3 (25.0%)	0.276
LDH_max_ (U/l) before ECMO	734.1 ± 268.2	577.2 ± 182.1	812.5 ± 275.5	0.083
Scores				
SOFA score	9.7 ± 3.6	8.7 ± 3.4	10.3 ± 3.7	0.733
APACHE II score	24.9 ± 8.1	25.0 ± 9.0	24.9 ± 8.1	0.961
RESP score	− 3.3 ± 3.2	− 2.8 ± 1.9	− 3.55 ± 3.8	1.0
Successful ECMO weaning	7 (38.9%)	3 (50%)	4 (33.3%)	0.494
Survival^†^	4 (22.2%)	3 (50%)	1 (8.3%)	0.045
ICU length of stay (days)	26.2 ± 20.5	33.8 ± 15.4	22.4 ± 22.3	0.053
ECMO duration (days)	13.2 ± 8.7	13.8 ± 11.0	12.9 ± 7.8	0.892
MV duration (days)	20.8 ± 14.8	25.2 ± 17.1	18.4 ± 13.7	0.462
Acute haemodialysis	6 (33.3%)	0 (0%)	6 (50.0%)	0.054
Prone position while ECMO	11 (61.1%)	5 (83.3%)	6 (50.0%)	0.588

*ICU* intensive care unit, *MV* mechanical ventilation

*Underlying pulmonary disease: lung fibrosis (*n* = 2)

^†^ICU and hospital survival

**Fig. 1:**
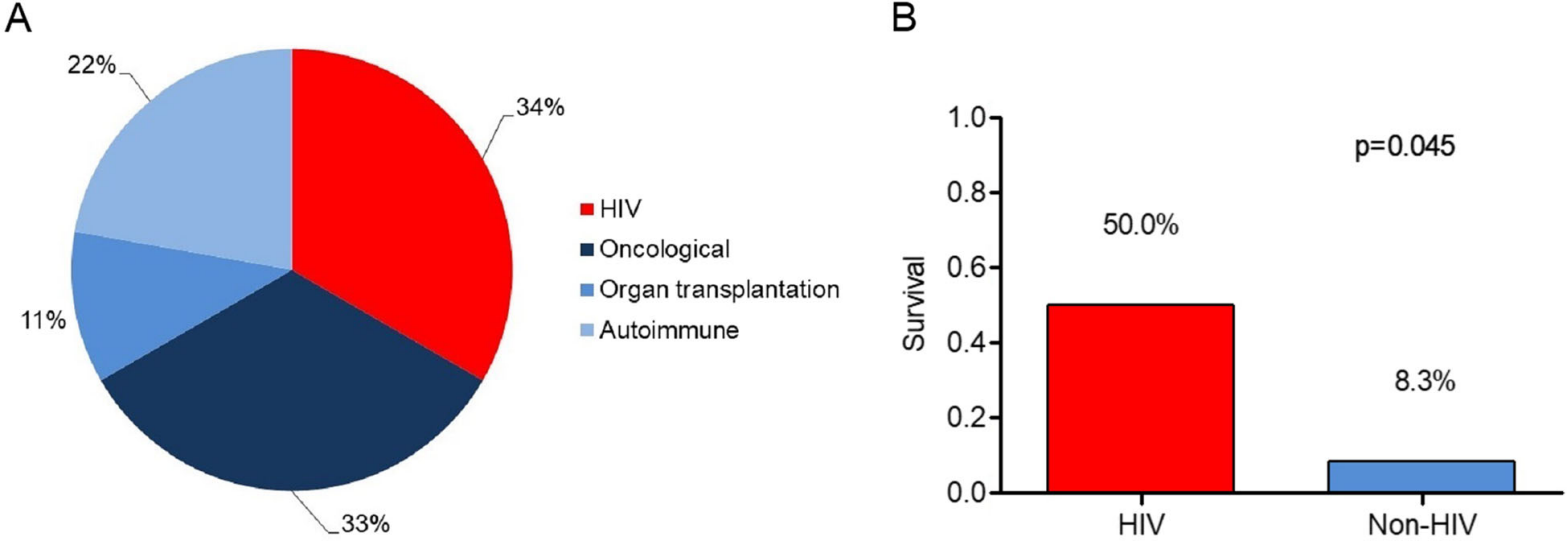
**a** Underlying subtypes of immunosuppression in ECMO patients with PJP. **b** Survival of PJP with severe respiratory failure and ECMO therapy in HIV vs. non-HIV patients

HIV was the cause of immunosuppression in 6 patients, whereas 12 patients had other subtypes of immunosuppression (non-HIV group, Fig. 1a). In all cases, HIV was diagnosed during index hospitalisation. Patients therefore were without previous antiretroviral treatment.

There were no significant differences between these two groups in relation to sex, comorbidities, ventilator settings, LDH levels or survival prediction scores (SOFA, APACHE II and RESP, Table 1). Patients with HIV were younger than non-HIV patients, and the interval between the start of mechanical ventilation and ECMO therapy was shorter in the non-HIV group.

Overall ECMO weaning rate was 39%, without a significant difference between HIV and non-HIV patients. Overall hospital survival was 22%. Withdrawal of care when further curative treatment was deemed futile was the most common cause of death (nine patients, 64.3%). Survival rate was higher in HIV than in non-HIV patients (50% vs. 8%, *p* = 0.045, Fig. 1b).

It has been shown previously in a non-ECMO setting that the outcome in HIV-negative PJP patients is worse than in patients with HIV [3], and our data confirm these earlier observations.

There are possible explanations for the better prognosis of HIV in this setting. On average, HIV patients are younger, and immunosuppression in HIV patients is reversible and can be resolved with the initiation of antiretroviral treatment. Moreover, the high mortality of non-HIV patients is associated with the underlying disease itself and a faster and more fulminant progression of the disease with more severe hypoxia and a higher prevalence of shock [4].

One third of our patients in the non-HIV group could be weaned successfully from ECMO support, suggesting that mortality was not only associated with ARDS, but underlying comorbidities may have been predominant. Moreover, there was a trend towards more frequent acute haemodialysis in non-HIV patients, illustrating that these patients had more complications and suffered from multi-organ failure.

In summary, a survival rate of 50% in HIV patients is similar to the average survival of ECMO patients with ARDS of any origin as shown by the CAESAR (63%) or the EOLIA trial (65%) [5, 6]. Therefore, ECMO therapy should not be withheld from patients with HIV-associated PJP.

## Data Availability

The datasets used and/or analysed during the current study are available from the corresponding author on reasonable request.

## Abbreviations

APACHE II: Acute Physiology and Chronic Health Evaluation
ARDS: Acute respiratory distress syndrome
HIV: Human immunodeficiency virus
ICU: Intensive care unit
MV: Mechanical ventilation
PJP: *Pneumocystis jirovecii* pneumonia
RESP: Respiratory Extracorporeal Membrane Oxygenation Survival Prediction
SOFA: Sequential Organ Failure Assessment
VV-ECMO: Veno-venous extracorporeal membrane oxygenation

## References

[1] Schmidt JJ, Lueck C, Ziesing S, Stoll M, Haller H, Gottlieb J (2018). Clinical course, treatment and outcome of Pneumocystis pneumonia in immunocompromised adults: a retrospective analysis over 17 years. Crit Care.

[2] Roux A, Canet E, Valade S, Gangneux-Robert F, Hamane S, Lafabrie A (2014). Pneumocystis jirovecii pneumonia in patients with or without AIDS. France Emerg Infect Dis.

[3] Monnet X, Vidal-Petiot E, Osman D, Hamzaoui O, Durrbach A, Goujard C, Miceli C, Bouree P, Richard C (2008). Critical care management and outcome of severe Pneumocystis pneumonia in patients with and without HIV infection. Crit Care.

[4] Salzer HJF, Schafer G, Hoenigl M, Gunther G, Hoffmann C, Kalsdorf B, Alanio A, Lange C (2018). Clinical, diagnostic, and treatment disparities between HIV-infected and non-HIV-infected immunocompromised patients with Pneumocystis jirovecii pneumonia. Respiration..

[5] Peek GJ, Mugford M, Tiruvoipati R, Wilson A, Allen E, Thalanany MM (2009). Efficacy and economic assessment of conventional ventilatory support versus extracorporeal membrane oxygenation for severe adult respiratory failure (CESAR): a multicentre randomised controlled trial. Lancet..

[6] Combes A, Hajage D, Capellier G, Demoule A, Lavoue S, Guervilly C (2018). Extracorporeal membrane oxygenation for severe acute respiratory distress syndrome. N Engl J Med.

